# Who can help me? Understanding the antecedent and consequence of medical information seeking behavior in the era of bigdata

**DOI:** 10.3389/fpubh.2023.1192405

**Published:** 2023-09-18

**Authors:** Jiwei Sun, Shujie Zhang, Min Hou, Qian Sun, Fenglin Cao, Zhonghao Zhang, Guiyao Tang, Xingyuan Wang, Ling Geng, Linlin Cui, Zi-Jiang Chen

**Affiliations:** ^1^Center for Reproductive Medicine, Cheeloo College of Medicine, Shandong University, Jinan, China; ^2^Research Unit of Gametogenesis and Health of ART-Offspring, Chinese Academy of Medical Sciences, Jinan, China; ^3^Key Laboratory for Reproductive Endocrinology, Ministry of Education, Shandong University, Jinan, Shandong, China; ^4^Shandong Key Laboratory of Reproductive Medicine, Jinan, China; ^5^Shandong Provincial Clinical Medicine Research Center for Reproductive Health, Jinan, Shandong, China; ^6^Shandong Technology Innovation Center for Reproductive Health, Jinan, China; ^7^National Research Center for Assisted Reproductive Technology and Reproductive Genetics, Jinan, China; ^8^School of Nursing and Rehabilitation, Cheeloo College of Medicine, Shandong University, Jinan, Shandong, China; ^9^Business School, Shandong Normal University, Jinan, Shandong, China; ^10^School of Management, Shandong University, Jinan, Shandong, China; ^11^Shandong Provincial Hospital Affiliated to Shandong First Medical University, Jinan, China; ^12^Shanghai Key Laboratory for Assisted Reproduction and Reproductive Genetics, Shanghai, China; ^13^Center for Reproductive Medicine, Renji Hospital, School of Medicine, Shanghai Jiao Tong University, Shanghai, China

**Keywords:** medical information seeking, satisfaction with information, information overload, patient-physician relationship, stress coping theory, information processing theory

## Abstract

**Introduction:**

The advent of bigdata era fundamentally transformed the nature of medical information seeking and the traditional binary medical relationship. Weaving stress coping theory and information processing theory, we developed an integrative perspective on information seeking behavior and explored the antecedent and consequence of such behavior.

**Methods:**

Data were collected from 573 women suffering from infertility who was seeking assisted reproductive technology treatment in China. We used AMOS 22.0 and the PROCESS macro in SPSS 25.0 software to test our model.

**Results:**

Our findings demonstrated that patients’ satisfaction with information received from the physicians negatively predicted their behavior involvement in information seeking, such behavior positively related to their perceived information overload, and the latter negatively related to patient-physician relationship quality. Further findings showed that medical information seeking behavior and perceived information overload would serially mediate the impacts of satisfaction with information received from physicians on patient-physician relationship quality.

**Discussion:**

This study extends knowledge of information seeking behavior by proposing an integrative model and expands the application of stress coping theory and information processing theory. Additionally, it provides valuable implications for patients, physicians and public health information service providers.

## 1. Introduction

With the rapid development and growing prevalence of internet based information technology, the internet is becoming the primary tool for patients in search of medical information (1–3). According to Pew Internet Project’s research, over 80% of internet users in the United States search for health information online (4). Along with the increase in online health information search, the volume of medical related information on the internet is surging (1). Medical information has flooded the internet and penetrated daily life through computers, mobile phones, and other media (2, 5). The internet provides new ways of transmitting medical information in a convenient manner (6), but also brings potential risks that cannot be ignored (7–9).

In the context of health care, when patients receive a diagnosis from their physicians, the process of coping with uncertainty would be triggered (10). The typical response of patients is to search for a frame of reference that enables them to assess the severity of their condition (10). For example, they may ask their physicians “Am I in danger? Will I be okay? How bad is this?” When patients suspect that the physician does not provide accurate answers to their questions or is holding something back from them, and they are dissatisfied with the information provided by their physicians, they may experience the accumulation of uncertainty (10). To reduce the perceived stress caused by uncertainty, seeking information from other sources can be used to enhance coping by helping individuals understand the health threat and the its associated challenges (11), determine available resources to manage the stressors, and thus increase predictability and feelings of control (12–14).

However, exposure to excessive amounts of medical information may lead to information overload (1, 15, 16), where an individual’s efficiency in using available information is hampered by its overwhelming quantity (17). According to the information processing theory, the cognitive resources for an individual to select, store and retrieve information are limited (15, 18). Receiving a high variety of information requires patients to identify the most useful parts related to their symptoms, diagnosis, treatment and so on (1). Unfortunately, it is difficult for patients with limited medical knowledge to filter the important information and separate it from noise (15, 19). When new information continuously arrives and competes for limited processing resources, patients may experience strain in their capacity to process information (17, 18). Just as Jiang and Beaudoin claimed, information overload can result from people’s continued efforts in searching for information (16).

Information overload can cause various adverse effects on patients’ cognition, emotions, and attitudes (1, 5, 20). Eppler and Mengis reported that patients experiencing information overload tend to feel stressed and confused, and ignore further information input (18). In addition, the research conducted by Swar et al. has shown that perceived information overload is positively associated with negative affect, depressive symptoms, and feelings of anxiety and anger (1). The change in patients’ psychological state and emotion may affect their interaction with physicians, and subsequently influence the quality of their relationship. In line with this perspective, a study examined the effects of information overload on patients’ behavioral intentions and suggested that perceived information overload had a direct negative impact on patients’ compliance in treatment (5).

**Figure 1:**
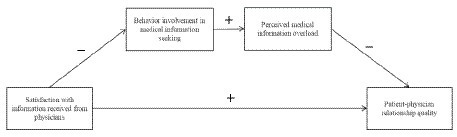
Integrate model of medical information seeking. In our model, we propose that: (1) patients’ satisfaction with the information received from physicians is negatively related to their involvement in medical information seeking. (2) Behavior involvement in medical information seeking is positively related to perceived information overload. (3) Perceived information overload is negatively related to patient-physician relationship quality. (4) Behavior involvement in medical Information seeking and perceived information overload play a sequential mediating role in the relationship between patients’ satisfaction of information received from physicians and patient-physician relationship quality.

Echoing this trend, we proposed an integrated theoretical model that encompasses the antecedent (i.e., satisfaction with information received from physicians) and consequences (i.e., medical information overload and patient-physician relationship quality) of medical information seeking. In terms of research subjects, we focus on women suffering from infertility. In recent years, information on assisted reproduction has grown rapidly. The explosion of such information, however, has greatly disturbed the normal diagnostic and treatment procedures for women suffering from infertility due to their difficulty in distinguishing the quality of relevant information, which may lead to conflict and confusion. Therefore, we target women suffering from infertility as the research subjects of this study and explore the antecedents and outcomes of their information seeking behavior. Specifically, we propose that patients’ satisfaction with the information received from physicians is negatively related to their involvement in medical information seeking, which may result in perceived information overload. Consequently, patients’ perception pf information overload may undermine the quality of their relationship with physicians. Additionally, we assume that information seeking and information overload play a sequential mediating role in the relationship between patients’ satisfaction of information received from physicians and quality of their relationship with their physicians. In summary, Figure 1 presents our conceptual model.

## 2. Methods

### 2.1. Participants and procedure

We contacted a hospital for reproductive medicine in China to collect data. This hospital is one of the largest reproductive hospitals in eastern China. The patients at this hospital have diverse demographic backgrounds, including various age groups and different socioeconomic statuses, which enables us to obtain reliable and generalizable research results. Prior to conducting our field survey, we contacted the managers in charge of this hospital and clearly communicated that our project was intended solely for research purposes and strictly confidential. After gaining approval for the study, we printed questionnaires and sent them to doctors in charge of the assisted reproductive technology (ART) unit who helped us in distributing these questionnaires to all voluntary participants.

The sample consisted of women who arrived for their initial visit at the ART units in this hospital. Women were included in the study if they were in the first phase of fertility consultation and could understand and complete questionnaires in Chinese. We placed no restrictions on age, education, or socioeconomic status. The doctors in charge of the unit approached the participants and introduced the broad topic of the study as well as its requirements for participation. Subsequently the doctors asked if they would like to participate in the study. To encourage participation, they assured participants that (1) their participation will be voluntary, (2) surveys will be kept confidential, (3) they have right to retrieve and/or withdraw their information from the study at any time, (4) their response will be used for academic research purposes only, and (5) their participation was in no way related to the medical treatment they would receive at the clinic. With the written informed consent of the participants, the women received the questionnaire in a sealed envelope. Subsequently, the authors contacted the women to verify that the questionnaire had been completed and submitted, and to respond to any distress it may have aroused. After completed the questionnaires, the participant received a letter of thanks from us for his/her contribution to the study.

During the data collection, 602 women met the inclusion criteria (i.e., they were in the initial phase of ART and were capable of completing the Chinese instruments). Twenty-nine women declined to participate. The final sample consisted of 573 women aged 22–47 (M = 33.86, SD = 4.91), Table 1 shows the demographic characteristics of the 573 participants.

**Table 1:** Demographic characteristics of the participants (*n* = 573).

Variables	*N*	%
Age		
<30	145	25.31
30 ~ 34	256	44.68
35 ~ 39	118	20.59
≥40	54	9.42
Education		
9th grade and below	159	27.74
High school	118	20.59
Junior college	123	21.47
Bachelor’s degree	134	23.39
Master’s degree and above	34	5.93
Did not respond	5	0.87
Monthly income (Yuan)		
<1,500	79	13.79
1,500 ~ 3,000	149	26.00
3,000 ~ 5,000	197	34.38
5,000 ~ 8,000	61	10.65
8,000 ~ 10,000	24	4.19
>10,000	12	2.09
Did not respond	51	8.90

### 2.2. Instruments

We translated the measures from English to Chinese following Brislin’s (21) translation-back translation procedure. All ratings were made *via* a 5-point Likert scale ranging from 1 (strongly disagree) to 5 (strongly agree) unless otherwise indicated. To evaluate the internal consistency reliability of the scales, we calculated Cronbach’s alpha coefficient.

#### 2.2.1. Satisfaction with information received from physician

A 3-item scale, adapted from Matthews et al. (22), was used to assess the woman’s subjective satisfaction with information provided by their physicians (e.g., “I am satisfied with the information I received from my primary physician(s) about my diagnosis”). Since the original scale developed by Matthews et al. was used to assess cancer patients’ satisfaction with the medical information received from their physicians (22), we modified some of these statements to suit our research context of assisted reproductive technology. Cronbach’s alpha value for this scale in this study was 0.90. The mean score was computed by averaging the responses to all three items, with higher scores indicating greater satisfaction with the information provided by physicians.

#### 2.2.2. Behavior involvement in medical information seeking

Behavior involvement in medical information seeking was rated by the 9-item adapted from Krantz et al. (23). Example items include “I tend to learn how to cure some of my own illness without contacting a physician.” Cronbach’s alpha for this scale in this study was 0.68. The mean scores were computed for each participant by averaging the responses to all items, with higher scores indicating greater level of involvement in information seeking behavior.

#### 2.2.3. Perceived information overload

We measured participant’s perceived information overload using a 13-item scale adapted from Jensen et al. (24). Sample items are “There are so many different recommendations about assisted reproductive technology, it’s hard to know which ones to follow” and “It has gotten to the point where I do not even care to hear new information about assisted reproductive technology.” Cronbach’s alpha for this scale in this study was 0.92. The mean scores were computed for each participant by averaging the responses to all items, with higher scores indicating greater perception of information overload.

#### 2.2.4. Patient-physician relationship quality

We used a 5-item scale, which was adapted from Ganz et al. (25), to measure the women’s perceived relationship quality with her provider (e.g., “It’s difficult to discussing new symptoms with my doctors”). Cronbach’s alpha for this scale in this study was 0.89. The mean total score was computed by averaging the responses to all items, with a higher score indicating the lower quality of patient-provider relationship.

#### 2.2.5. Demographic questionnaire

A demographic questionnaire was used to obtain information regarding personal characteristics, including age, education and monthly income. Detailed information was present in Table 1.

### 2.3. Data analysis

IBM SPSS 25.0 and AMOS 22.0 software was used for statistical analysis. First, prior to the main analysis, we conducted several preliminary analyses. Specifically, we used SPSS 25.0 to address the issue of missing values due to incomplete questionnaires since it may cause biased sampling. Analysis across the core variables revealed relatively low rates of missing values, ranging from as low as none to a high of 5.2 percent. Little’s test for Missing Completely at Random (MCAR) revealed that missing items were completely at random (*χ*^2^ = 1689.118, *df* = 1,652, *p* = 0.257), and that no missing values were related to a specific variable or a specific respondent (26).

In the second stage, common method bias was tested followed the suggestion of Podsakoff et al. (27). Besides, descriptive analyses were conducted to calculate the means, standard deviations of core variables and Pearson correlation was used to examine the associations between variables. Composite reliabilities and average variance extracted were also calculated in this stage. Next, a series of confirmatory factor analysis was conducted in AMOS 22.0 to assess the measurement model. Finally, we adopted the PROCESS macro in SPSS 25.0 software with bootstrapping techniques developed by Preacher and Hayes (28) to test our hypotheses. In light of the literature, background characteristics (age, education, income) were entered as control variable in the model (2, 29).

## 3. Results

### 3.1. Tests of common method bias

We used Harman’s single factor procedure to address the issue about common method bias raised by the measures we used. The logic underlying this approach is that if method variance is largely responsible for the covariation among the measures, a factor analysis should yield a single (method) factor (27). Therefore, principal component analysis without rotation was conducted. The statistical results show that there are 5 factors whose eigenvalues are greater than 1, and the first factor accounts for 29.27% of the total variance, which is far lower than the critical value of 40%. These suggest that common method bias did not cause a serious threat to interpreting our findings.

### 3.2. Descriptive statistics

Table 2 shows the mean scores and standard deviations of variables. The results of Pearson correlations are also presented in Table 2. The results reveal that women’s satisfaction with information has significant negative relationships with behavior involvement in information seeking (*r* = −0.28, *p* < 0.001). This indicts that patients with lower satisfaction have more information search behavior. Besides, there is a positive correlation between behavior involvement in information seeking and perceived information overload (*r* = 0.14, *p* < 0.01). Thus, higher behavior involvement in information seeking is associated with higher perceived information overload. In addition, perceived information overload (*r* = −0.51, *p* < 0.001) is negatively associate with perceived patient-provider relationship quality. It implies that information overload may undermine patient-provider relationship.

**Table 2:** Mean, standard deviation, and correlation between variables.

Variables	M	SD	1	2	3	
Satisfaction with information	4.13	0.67	**0.71**			
Behavior involvement in information seeking	2.19	0.46	−0.28^***^	**0.71**		
Perceived information overload	2.81	0.78	−0.24^***^	0.14^**^	**0.72**	
Patient-physician relationship quality	3.71	1.06	0.44^***^	−0.26^***^	−0.51^***^	**0.82**

The diagonal elements (in bold) represent the square root of AVE.

### 3.3. Psychometric properties

Table 3 shows the assessment of composite reliabilities and convergent validity. Composite reliabilities (CR) in the proposed model are above the 0.7 threshold indicating a high reliability of items used for each construct. Convergent validity is assessed by evaluating the average variance extracted (AVE) from the measures. The AVE is above the threshold value of 0.5, meeting the criteria of convergent validity. Discriminant validity is assessed by examining the square root of AVE as recommended by Fornell and Bookstein (30). As shown in Table 2, the square root of AVE of each construct is greater than the correlations between itself and all other constructs. Moreover, all the constructs are found to have a stronger correlation with their own measures than to those of others. This also shows the proper assessment of discriminant validity.

**Table 3:** Psychometric properties of the measurement model.

Variables	CR	AVE
Satisfaction with information	0.88	0.67
Behavior involvement in information seeking	0.90	0.50
Perceived information overload	0.93	0.51
Patient-physician relationship quality	0.84	0.52

CR, composite reliability; AVE, average variance extracted.

Next, a series of confirmatory factor analyses (CFAs) was conducted. We used AMOS 22.0 to conduct the CFAs by contrasting the four-factor CFA model against alternatives to evaluate the distinctiveness of the key variables. As can be seen in Table 4, the four-factor model (including all factors we hypothesis) fits the data considerably better than any of the alternatives (*χ^2^* (371) = 1131.116, *p* < 0.001; Comparative Fit Index (CFI) = 0.910, Tucker-Lewis Index (TLI) = 0.905, Root Mean Square Error of Approximation (RMSEA) = 0.060, Akaike information criterion (AIC) = 1259.116, Bayesian Information Criterion (BIC) = 1537.572).

**Table 4:** Results of confirmatory factor analysis for variables studied.

Model	*χ* ^2^	*df*	CFI	TLI	RMSEA	AIC	BIC
Four-factor model	1131.116	371	0.910	0.905	0.060	1259.116	1537.572
Three-factor model-1	1852.891	374	0.814	0.798	0.083	1974.891	2240.295
Three-factor model-2	2204.759	403	0.777	0.759	0.088	2328.759	2598.514
Three-factor model-3	2352.906	402	0.759	0.739	0.092	2478.906	2753.012
One-factor model	4233.147	405	0.526	0.491	0.129	4353.147	4614.201

Three-factor model-1: satisfaction with information and behavior involvement in information seeking combined; Three-factor model-2: behavior involvement in information seeking and perceived information overload combined; Three-factor model-3: perceived information overload and patient-physician relationship quality combined. CFI, comparative fit index; TLI, Tucker-Lewis index; RMSEA, root-mean-square error of approximation; SRMR, standard root mean-square residual; AIC, Akaike information criterion; BIC, Bayesian information criterion.

### 3.4. Tests of hypothetical model

In the next stage, the PROCESS macro in SPSS 25.0 software was used to test our hypothesis model. The results in Table 5 reveal that, patients’ satisfaction of information received from physicians has a negative and significantly effect on behavior involvement in medical information seeking (*β* = −0.19 s.e. = 0.03, *p* < 0.001, 95% CI = [−0.25, −0.14]). In addition, patients’ behavior involvement in medical information seeking has a significantly positive effect on perceived medical information overload (*β* = 0.14, s.e. = 0.07, *p* < 0.05, 95% CI = [0.01, 0.29]). Besides, the result shows that the relationship between perceived medical information overload and patient-physician relationship quality is negative and significant (*β* = −0.27, s.e. = 0.08, *p* < 0.001, 95% CI = [−0.42, −0.11]), indicating that the more a woman perceives information overload, the worse quality of the relationship with her physicians she experienced.

**Table 5:** Results of hypothetical model.

	Information seeking	Information overload	patient-physician relationship quality
Age	−0.00	0.01^*^	−0.00
Education	−0.01	−0.03	0.10^***^
Monthly income	−0.01	−0.02	−0.02
Satisfaction with information	−0.19^***^	−0.26^***^	0.48^***^
Information seeking		0.14^**^	−0.33^***^
Information overload			−0.27^***^
	Effect	SE	95% CI
Direct effect of SI on PPRQ	0.49	0.06	[0.38, 0.60]
Indirect effect of SI on PPRQ through	0.02	0.03	[0.01, 0.03]

SI, satisfaction with information; PPRQ, patient-physician relationship quality. Bootstrap samples = 5,000; 95% CI: 95% confidence intervals. ^***^p < 0.001, ^**^p < 0.01, ^*^p < 0.05. SE, standard error.

To further verify the indirect or mediated effect of information seeking and information overload, we use the 95% bias-corrected bootstrapped confidence intervals (CI) provided by Preacher and Hayes (28). Bootstrapping is a ‘nonparametric’ way of computing a sampling distribution, which has been recommended as a more powerful method of testing conditional indirect effect (28). As the results of bootstrapping showed, the direct effect of satisfaction of information on patient-physician relationship quality is significant (*β* = 0.49, s.e. = 0.06, 95% CI = [0.38, 0.60]), and the indirect effect of satisfaction of information on patient-physician relationship quality through information seeking and information overload (*β* = 0.02, s.e. = 0.03, 95% CI = [0.01, 0.03]) is also significant.

## 4. Discussion

With the expanding availability of medical and health information, more and more patients tend to search for and acquire relevant information from multi-source by themselves (2, 3). Since topics related to medical information seeking are emerging but underestimated, this study focuses on the patients’ involvement in seeking medical information and examines the relationship between patients’ satisfaction with information received from physicians, information seeking behavior, perceived information overload, and the quality of their relationship with their physicians.

Specifically, we explore the relationship between perceived information satisfaction and medical information seeking behavior by drawing on stress coping theory. In addition, based on information processing theory, the relationships between medical information seeking behavior, information overload and patient-practitioner relationship quality are investigated. Next, we examine the serial mediating effect of information seeking and information overload on the relationship between satisfaction with information and patient-practitioner relationship quality.

The results of our study show that the patients’ mistrust of their practitioners may lead to information seeking behavior. With the increasing amount of varied information encountered, patients may experience information overload. As patients typically do not possess deep prior knowledge of the symptoms, diagnosis, treatment or administration of their health conditions, it is difficult for them to filter and separate useful information from large volume of noise (1, 2, 15, 16). Thus, information overload may damage the relationship between patients and practitioners, which further lead to conflicts in medication choices and other treatment issue.

### 4.1. Theoretical contributions

Medical information seeking shows how people assessing the medical information needs, partnering with other medical information resources and acting on information transmitted to them from various information carriers (31). Few of studies have investigated the role of the interact between patients and physicians might play in predicting the information seeking behavior, and little is known about the dark-side effects of information seeking on the patients themselves and the relationship with their physicians (2, 5). Our study is one of the few studies to explore the medical information seeking behavior of a Chinese sample. By theoretically constructing and empirically testing a synthetic model that integrates the factors that influence information seeking behavior and the potential dark-side effect of such behavior. Our research contributes to the literature of medical information seeking.

First, our research explores the important role of information satisfaction plays in predicting information seeking behavior. Although past research has indicated there are many predictors of information seeking, most of them focus on the individual characteristics (e.g., socioeconomic status, information seeking preferences and experiences) (32, 33), little is known about the influence of information lacking on the seeking behavior of patients. By involving stress coping theory, our study demonstrate that patients’ satisfaction of information received from their physicians impacts their behavior involvement in medical information seeking.

These findings expand the application of stress coping theory and indicate that medical information seeking can be used as a coping strategy for patients who lacking necessary information to fulfils their needs for control the stressful situation (2, 10, 11). According to stress coping theory, when encounter health problems, individual choose the next coping strategy according to the information he/she already has (10, 11). For patients who dissatisfied with information provided by their physicians (such as have unanswered questions about their illness and treatment), their feeling of stress caused by uncertainty increase, which compel them to seek additional information through information channels other than physicians.

Second, our study responds to the call of in-depth research on the outcomes of information seeking (16, 34, 35) and also expands the application of information processing theory into a new field. The findings demonstrate that the information seeking behavior can cause perceived information overload and further damage the relationship between patients and physicians. In addition, the results of our study reveal that the satisfaction of information can affect patient-physician relationship quality *via* the mechanism of behavior involvement in information seeking and perceived information overload.

Consistent with information process theory, these results suggest that with the limitation of cognitive resources, information seeking can lead to the threat of information overload. With the deepening of information seeking, the information volume and heterogeneity increase and information relevance decrease (36), which bring heavy burden to individual’s cognitive resources and increase the possibility of information overload (1, 15, 16). Excessive and diverse information may interfere with the process of information filtering, selecting and processing (3), which trigger changes in patients’ cognition, emotion and attitude (3, 5, 20) and impact the patient-physician relationship quality.

### 4.2. Practical implications

By considering the complexity and cognitive aspects of information seeking, this study provides important implications for public health promotion, patient empowerment, and quality of health communication. It also bring a good opportunity for health information professionals to contribute more to this interdisciplinary discourse. First of all, our study describes the causes of patients’ anxiety about excessive information and implies a solution to the physician-patient communication. It is reasonable to assume that the informational support provided by their physicians might help patients to cope with and prevent information overload. Studies on the use of and preferences for information sources among health information seekers show that there is a discrepancy between the sources patients reported to have used (that is, the Internet) and the sources they preferred to use (that is, health care providers) (19). In line with this, our research suggests that it is better for professionals to provide more health related information to public and undertake the responsibility of patient education.

Secondly, it is suggesting for patients to monitor their information seeking behavior and thus protect themselves from drowning in information. As researches indicated, confusion might increase as the number of sources increase and particular sources may not be in a good position to make any type of relevance judgments, nor is it guaranteed that they can evaluate the quality of the information accurately (1, 15, 16). So, we suggest that patients can take the “usefulness of information” as the main criterion when they receive massive information, and break the habit of labelling a large amount of information as “probably useful.” It is also important for patients to improve their information literacy, which includes the ability to discover, evaluate, use and exchange information (35). In turn, this underscores the importance of literacy approaches in health communication and education campaigns (37).

Thirdly, this empirical research on medical information overload brings us closer to improving information campaigns and services to help individuals with different literacy levels meet their specific health information needs. During the information exchange between the proxies and the patient, some information might not be communicated accurately or completely, which may result lead to confusion (16, 34). Therefore, it is suggested that information service providers to update and strengthen the function of information purification/classification, make statistical analysis of the terms in the web searched by users, and classify the search result according to the subject matter, so as to save users’ energy in viewing a large number of websites.

In sum, due to the expanding availability of medical and health information, it is difficult to curb the patients’ tendency to seek medical information. The informational support provided by the physicians could help patients to cope with and prevent information overload. It was suggested that medical professionals should undertake the responsibility of patient education and provide more health related information to the public. Furthermore, information service providers should update and strengthen the function of information purification/classification, so as to save users’ energy from the web noises.

### 4.3. Limitations and future research

There are several limitations of this study which may provide inspirations for future research. First, although we provide an integrated model of medical information seeking, the mechanisms underlying the relationships among variables need further examination. For example, future research can focus on how information overload affects patient-physician relationship and explore the mediation role of cognitive resource depletion, negative emotion etc., and the moderation role of social support, self-regulation and other alternative factors. In addition, this study has examined the effects of information seeking behavior on information overload and patient-physician relationship, further examination of other outcomes (such as clinical compliance and self-treatment) of medical information seeking may help to draw a more comprehensive picture of medical information seeking.

We recognized that information seeking is a complex and context based construct, and we acknowledge that relying solely on self-reported data clearly limits our ability to make any clear cut generalizations from our findings. This as an important opportunity to explore and identify the essence part of medical information seeking behavior for future studies. For example, by collecting real time data on the web, future studied can analysis the differences of user information seeking behavior under various search situations.

For health information professionals, this research brings important questions to the fore. What are the characteristics of people who suffer from medical information overload? How to protect patients from information overload? What patients do to cope with overload? The answers to these questions have important implications on how we should deliver health information and assess future information services. Therefore, future research can use experimental method or long-term surveys to explore the above issues and propose relevant interventions.

## Data availability statement

The raw data supporting the conclusions of this article will be made available by the authors, without undue reservation.

## Author contributions

SZ, JS, and GT: conceptualization. SZ, JS, and LG: data curation. SZ and QS: formal analysis. LC: funding acquisition. JS, MH, ZZ, LG, and LC: investigation. SZ, JS, QS, FC, GT, and XW: methodology. MH: project administration. Z-JC: resources. GT, LC, and Z-JC: supervision. SZ and JS: writing – original draft. QS, FC, and XW: writing – review and editing. All authors contributed to the article and approved the submitted version.

## Funding

This study was supported by the National Key R&D Program of China (2022YFC2702905), CAMS Innovation Fund for Medical Sciences (2021-I2M-5-001), Taishan Scholars Program for Young Experts of Shandong Province (tsqn201909195), National Natural Science Foundation of China (32200897, 72072101), and Shandong Provincial Natural Science Foundation (ZR2021QC147). The funders had no role in study design, data collection and analysis, decision to publish, or preparation of the manuscript.

## Conflict of interest

The authors declare that the research was conducted in the absence of any commercial or financial relationships that could be construed as a potential conflict of interest.

## Publisher’s note

All claims expressed in this article are solely those of the authors and do not necessarily represent those of their affiliated organizations, or those of the publisher, the editors and the reviewers. Any product that may be evaluated in this article, or claim that may be made by its manufacturer, is not guaranteed or endorsed by the publisher.
